# Negative Regulation of NF-κB by the ING4 Tumor Suppressor in Breast Cancer

**DOI:** 10.1371/journal.pone.0046823

**Published:** 2012-10-04

**Authors:** Sara A. Byron, Elizabeth Min, Tanya S. Thal, Galen Hostetter, Aprill T. Watanabe, David O. Azorsa, Tanya H. Little, Coya Tapia, Suwon Kim

**Affiliations:** 1 Cancer and Cell Biology Division, Translational Genomics Research Institute, Phoenix, Arizona, United States of America; 2 Integrated Cancer Genomics Division, Translational Genomics Research Institute, Phoenix, Arizona, United States of America; 3 Clinical Translational Research Division, Translational Genomics Research Institute, Phoenix, Arizona, United States of America; 4 Basic Medical Sciences, University of Arizona College of Medicine, Phoenix, Arizona, United States of America; University of Hawaii Cancer Center, United States of America

## Abstract

Nuclear Factor kappa B (NF-κB) is a key mediator of normal immune response but contributes to aggressive cancer cell phenotypes when aberrantly activated. Here we present evidence that the Inhibitor of Growth 4 (ING4) tumor suppressor negatively regulates NF-κB in breast cancer. We surveyed primary breast tumor samples for ING4 protein expression using tissue microarrays and a newly generated antibody. We found that 34% of tumors expressed undetectable to low levels of the ING4 protein (n = 227). Tumors with low ING4 expression were frequently large in size, high grade, and lymph node positive, suggesting that down-regulation of ING4 may contribute to breast cancer progression. In the same tumor set, we found that low ING4 expression correlated with high levels of nuclear phosphorylated p65/RelA (p-p65), an activated form of NF-κB (p = 0.018). Fifty seven percent of ING4-low/p-p65-high tumors were lymph node-positive, indicating a high metastatic tendency of these tumors. Conversely, ectopic expression of ING4 inhibited p65/RelA phosphorylation in T47D and MCF7 breast cancer cells. In addition, ING4 suppressed PMA-induced cell invasion and NF-κB-target gene expression in T47D cells, indicating that ING4 inhibited NF-κB activity in breast cancer cells. Supportive of the ING4 function in the regulation of NF-κB-target gene expression, we found that ING4 expression levels inversely correlated with the expression of NF-κB-target genes in primary breast tumors by analyzing public gene expression datasets. Moreover, low ING4 expression or high expression of the gene signature composed of a subset of ING4-repressed NF-κB-target genes was associated with reduced disease-free survival in breast cancer patients. Taken together, we conclude that ING4 negatively regulates NF-κB in breast cancer. Consequently, down-regulation of ING4 leads to activation of NF-κB, contributing to tumor progression and reduced disease-free patient survival in breast cancer.

## Introduction

Nuclear Factor kappa B (NF-κB) is a central molecule that mediates immune response by activating gene transcription. The canonical pathway of NF-κB activation involves receptor signaling leading to phosphorylation and proteasome-mediated degradation of Inhibitor of kappa B (IκB), resulting in the release of the NF-κB subunits from the cytoplasmic IκB complex. The NF-κB subunits, p65/RelA and p50/NF-κB1, then translocate into the nucleus where the p65/p50 heterodimers bind to target gene promoter sequences and activate transcription of a large number of genes including pro-inflammatory cytokines and chemokines, initiating the immune response [1], [2]. As acute as the NF-κB activation is, NF-κB is down-regulated by multiple mechanisms after initial immune response to prevent chronic inflammatory conditions that could lead to tissue damage and even death [2], [3]. In many cancers, NF-κB is constitutively active, resulting in elevated expression of NF-κB-target genes that elicit aggressive tumor cell behaviors including enhanced proliferation, survival, migration, invasion, metastasis, and therapy resistance [4], [5]. Thus, the molecular alterations that lead to constitutive activation of NF-κB pose a vital problem relating to cancer etiology and therapy.

In breast cancer, NF-κB activation has been better characterized in the human epidermal growth factor receptor 2-positive (HER2+) molecular subtype. Elevated DNA binding activity of NF-κB was found predominantly in HER2+ breast tumors [6]. *In vitro* studies have shown that the HER2/neu receptor could directly or indirectly activate the kinase cascade that results in the activation of NF-κB [7]–[9]. Moreover, inhibition of NF-κB by various genetic manipulations including the expression of IκBα or IκB kinase (IKK) mutants attenuated growth of HER2/neu receptor-initiated mammary tumors in MMTV-ErbB2/neu transgenic mice [10]–[12]. Therefore, these studies corroborated the role of HER2/neu signaling in NF-κB activation, which in turn contributes to the aggressive pathogenesis of HER2+ breast tumors. More recently, studies have shown that a subset of estrogen receptor-positive (ER+) breast cancers also contains elevated NF-κB activity associated with endocrine therapy resistance [13], [14]. In addition, a transcriptional synergy between estrogen receptor and NF-κB has been described, which results in a gene signature that correlates with chemo-resistance and poor patient outcome in a subset of ER+ breast cancer [15], [16]. While NF-κB activation in these ER+ breast tumors was partly attributed to HER2/neu receptor expression, other molecular mechanisms that lead to NF-κB activation in breast cancer are not well understood.

Inhibitor of Growth 4 (ING4) is a member of the ING tumor suppressor family and has been shown to play a role in many cancer-related cellular processes including cell proliferation, apoptosis, migration, angiogenesis, contact inhibition, DNA damage response, and hypoxia [17]–[24]. Gene deletion or reduced expression of *ING4* has been reported in various cancers including glioma, breast cancer, head and neck carcinoma, melanoma, hepatocellular carcinoma, gastric carcinoma, colon cancer, and lung cancer, implicating a tumor suppressive role of ING4 in diverse tissue types [20], [23], [25]–[32]. ING4 null mice, however, did not show increased spontaneous tumor formation, suggesting that ING4 deficiency alone may not be sufficient to initiate tumorigenesis [33]. We identified ING4 in a genetic screen for candidate tumor suppressors that could suppress loss of contact inhibition in tissue culture [23]. Subsequently, we showed that ING4 suppressed T47D breast cancer cell growth in soft agar and *MYC*-initiated mammary hyperplasia in a mouse model, providing evidence for the ING4 tumor suppressor function in breast cancer [23], [34]. Recently, we reported that 16.5% of breast tumors harbored an *ING4* gene deletion, suggesting a tumor suppressive role of ING4 in at least a subset of breast cancer [26].

Functionally, ING4 has been characterized as a transcription regulator with a mechanism involving chromatin remodeling. ING4 contains a plant homeodomain (PHD) finger motif conserved among the ING family members and other transcription factors [35], [36]. ING4 was shown to bind to tri-methylated histone H3 at lysine 4 (H3K4me3) via the PHD [37], [38]. In addition, ING4 was shown to co-purify with the HBO1/JADE histone acetyltransferase (HAT) complex, supporting a role of ING4 in chromatin modification [39], [40]. As H3K4me3 and HAT are generally associated with active transcription, ING4 was shown to activate gene transcription in response to DNA damage [17], [38]. In glioma and melanoma cancer models, however, ING4 repressed several NF-κB-target genes, thereby attenuating tumor angiogenesis and growth [20], [22], [25]. Whether the tumor suppressor function of ING4 involves the repression of NF-κB-target genes in other cancer types including breast cancer is currently unknown. Here, we present evidence that ING4 inhibits NF-κB in breast cancer cells and propose that down-regulation of ING4 is one of the molecular events that leads to NF-κB activation, promoting tumor progression and resulting in reduced patient survival in breast cancer.

## Materials and Methods

### Recombinant ING4 Protein Purification for Antibody Generation

DNA fragments encoding the N-terminal (AA 5–147) or C-terminal (AA 173–249) portion of ING4 were PCR-amplified using the pMIG-*ING4* construct [23] as a template with primer pairs: 5′-ATGTATTTGGAACATTATCTGGAC and 5′-CCCTTTGGAACGAGCACGAGC, 5′-ATGCCCTCAGTGACCTTTGGC and 5′- TTTCTTCTTCCGTTCTTGGGA. The DNA fragments were cloned into the pET21b bacterial expression vector (Novagen, Madison, WI) in the coding frame with a 6×HIS epitope-tag at the 3′ end of each fragment using *Eco*RI and *Xho*I restriction enzymes (New England BioLabs, Ipswich, MA). The DNA constructs were used to transform BL21 *E. coli* (Promega Corporation, Madison, WI). Recombinant proteins were induced by adding 1 mM IPTG (Promega) and purified from cell lysate using a Ni-NTA column (Novagen).

### Production of Monoclonal Antibodies to ING4

Monoclonal antibodies (mAbs) were generated as previously described [41] with the following modifications: Peptide ING4–156 corresponding to the ING4 amino acids 156–178 (CAPKTAQKKLKLVRTSPEYGMPS) was synthesized by Sigma-Genosys (The Woodlands, TX) with an additional cysteine at the N-terminus. The peptide was coupled to KLH and ovalbumin using a maleamide conjugation kit (Pierce Thermo Fisher Scientific, Rockford, IL). Female Balb/c mice (6–8 weeks old) were injected with an emulsion of 100 µg of recombinant ING4 or KLH-conjugated peptide ING4-156 with complete Freud’s adjuvant (Sigma-Aldrich, St. Louis, MO) by intraperitoneal injection 3 times at 2-week intervals, followed by injections of 75 µg of recombinant protein or peptide in PBS for three consecutive days. Splenocytes were isolated and fused to the myeloma cell line P3×63Ag8.653 using PEG:DMSO (50∶5, %v, Sigma-Aldrich). Fused cells were seeded in 96-well plates in DMEM:NCTC-109 (90∶10, %v, Invitrogen, Carlsbad, CA) media supplemented with 20% FBS (Invitrogen), 2 mM Glutamax I (Invitrogen), 25 mM Hepes, 1X HAT (Sigma-Aldrich), Penicillin/Streptomycin, and 0.5X Nutridoma-CS (Roche, Branchburg, NJ). Hybridoma colonies were screened by ELISA and were subcloned twice by limiting-dilution. Tissue culture supernatants from 6 independent hybridoma clones containing anti-ING4 mAbs termed BTIM-1 to BTIM-6, were collected and stored with 0.02% sodium azide at 4°C.

### Tissue Culture

The ING4 overexpression construct in the pMIG retroviral vector, pMIG-*ING4*, has been described previously [23]. The pMIG-*ING4HA* construct was generated by cloning the nucleotide sequence encoding the hemagglutanin (HA) epitope into the 3′ end of *ING4* via PCR. Cells were transfected with plasmids using Effectene (Qiagen, Valencia, CA). MCF10A, T47D, and MCF7 cells containing the pMIG vector or pMIG-*ING4* were generated using retroviral infection as described previously [23], followed by fluorescent activated sorting for green fluorescent protein-positive cells. Lentiviral particles containing a non-targeting shRNA construct (shNT) or ING4 knock-down construct (shING4) in the pLKO.1 vector (Sigma-Aldrich) were used to infect MCF10A cells. Cells containing shRNA constructs were selected in media containing 2 µg/ml puromycin (Sigma-Aldrich). The luciferase reporter plasmid, pGL4.32[*luc2*P/NF-κB/Hygro], was purchased from Promega Corporation. Cells containing pGL4.32[*luc2*P/NF-κB/Hygro] were selected in media containing 400 µg/ml hygromycin (Invitrogen). HEK293T cells were grown in DMEM containing 10% fetal bovine serum (FBS, Thermo-Fisher, Waltham, MA). T47D and MCF7 cells were grown in RPMI and MEM:EBSS media (Thermo-Fisher), respectively, supplemented with 10% FBS and 10 µg/ml bovine insulin (Sigma-Aldrich). MCF10A cells were grown in F10:DMEM media (Thermo-Fisher) supplemented with 10% FBS, 10 µg/ml bovine insulin, 10 ng/ml human epithelial growth factor (Invitrogen), and 1 µg/ml hydrocortisone (Sigma-Aldrich). Phorbol 12-myristate 13-acetate (PMA, Sigma-Aldrich) was dissolved in DMSO and used at a final concentration of 50 ng/ml.

### Immunofluorescent Staining and Western Blot Analysis

MCF10A and T47D cells containing various constructs were plated on chamber slides to 50% confluency, fixed with 4% paraformaldehyde, and permeabilized with 0.1% Triton X-100. Cells were immunostained with anti-ING4 antibody (BTIM-4, 1∶10 dilution) and Rhodamine Red X-conjugated donkey anti-mouse secondary antibody (1∶200, Jackson Immunoresearch, West Grove, PA). Cells were additionally stained for nuclei with 4′,6-Diamidino-2-phenylindole (DAPI, Vector Labs, Burlingame, CA) and visualized using a fluorescent microscope. For Western blot analyses, anti-ING4 antibody was used in 1∶10 dilution. Monoclonal antibodies for phospho-p65 (Ser536) (93H1) and p65/RelA (C22B4) and polyclonal antibodies for IκBα, IKKα, and IKKβ, were purchased from Cell Signaling and used at 1∶1,000 dilutions. Anti-tubulin (DM1A, 1∶2,000) and anti-histone H3 (1∶1,000) monoclonal antibodies were purchased from Millipore (Billerica, MA).

### Ethics Statement

Breast tumor samples were obtained from post-retention formalin-fixed and paraffin-embedded (FFPE) tissue blocks collected for clinical purposes between the years 1996 and 1999 at Banner Health (Phoenix, AZ). Tumor data regarding tumor size, histological subtype, grade, and TNM classification were obtained from de-identified pathology reports. All tissue samples and pathology report data were compiled adhering to the protocol (ghostetter05-018/05-0060-06) approved by Western Institutional Review Board (WIRB) and Banner Health Institutional Review Board. No informed patient consent was obtained because the samples had been collected for clinical purposes at the time of diagnosis and were retrieved post-retention retrospectively. The patient identity remained anonymous for this study. The Institutional Review Boards approved this waiver of patient consent. Additional FFPE blocks of breast tumor samples were obtained from Proteogenex (Culver City, CA), which declared that the samples were collected with informed patient consent under Institutional Review Board approved protocols (please see the full statement at http://www.proteogenex.com/AboutUs/EthicalIssues.html).

### Breast Tumor Tissue Microarray (TMA)

TMAs were constructed by extracting 0.6 mm diameter cores from the “donor” tumor tissue blocks and transferring tissue cores into a “recipient” paraffin block using an indexed manual arrayer, Tissue Arrayer VTA-100 (Veridiam Medical, El Cajon, CA) as previously described [42]. The TMAs contained 598 tissue punches from 249 independent tumor samples. Two hundred thirty and 351 tissue spots on TMAs represented “double” and “triple punches” from 115 and 117 tissue samples, respectively. A common set of normal breast tissue controls was included on each TMA.

### Immunohistochemistry (IHC)

Immunochemical staining of TMA sections was performed using BOND-MAX autostainer (Leica Microsystems, Germany). Antibodies used for IHC were anti-estrogen receptor-alpha (ER-α) monoclonal (1∶200, Novocastra, Newcastle Upon Tyne, UK), anti-HER2/neu polyclonal (1∶300, Novocastra), anti-phospho-p65 (Ser276) polyclonal (1∶40, Cell Signaling, Danvers, MA), and anti-ING4 monoclonal (1∶2, BTIM-4 cell culture supernatant). The staining intensity of estrogen receptor (ER) and HER2 was scored as described previously [43]. All TMA and whole sections were scored manually by a board certified pathologist (G.H). An IHC score was assigned to each sample by averaging the scores of double or triple punch samples. The percent of evaluable IHC staining on TMAs ranged from 94.5% (ING4) to 99% (p-p65).

### Cell Invasion Assay

Invasion assays were performed using 24-well chamber inserts with a basement membrane protein-coated polycarbonate membrane with 8 µm pores (Cell Biolabs, San Diego, CA). Three hundred thousand T47D-pMIG or T47D-ING4 cells were seeded in the upper compartment of the chambers with the medium containing 50 ng/ml PMA placed in the bottom compartment. After 48 hours, cells on the opposite side of the membrane were fixed in cold 70% methanol, stained with Hoechst stain (1∶1000, Invitrogen), and visualized using a fluorescence microscope. Cell numbers were determined by averaging cell numbers from a minimum of 4 field images per membrane.

### Luciferase Assay

Cells plated in a 24-well dish at 50% confluency were treated with or without 50 ng/ml PMA for 24 hours. Luciferase activity in 30 µls of cell lysates was measured using a Steady-Glo Luciferase Assay System (Promega Corporation) and Victor3 luminometer (Perkin Elmer Life Sciences Products, Boston MA). Total protein concentration in cell lysates was measured using the BCA Protein Assay Kit (Pierce Thermo Fisher Scientific). Luciferase activity was calculated as relative light units per microgram of protein and normalized to the *luc2* gene copy number integrated into the genome. *Luc2* gene copy number was determined relative to a pGL4.32[*luc2*P/NF-κB/Hygro] plasmid DNA standard curve using quantitative PCR (qPCR) with Fast SYBR Green Master Mix (Applied Biosystems, Foster City, CA) and two primer pairs specific to the *luc2* gene, (Forward-1 5′-TGTGTCCGATTCAGTCATGC-3′; Reverse-1 5′-TTGCTTAGGTCGTACTTGTCG-3′; Forward-2 5′-AGGCTACAAACGCTCTCATC-3′; Reverse-2 5′- ACATAGTCCACGATCTCCTTC-3′).

### Reverse Transcription Quantitative PCR (RT-qPCR)

Total RNA was isolated from 5×10^5^ cells using the RNeasy mini kit (Qiagen). Complementary DNA was synthesized from 2 µg of total RNA using the Superscript III First-Strand Synthesis SuperMix kit with oligo dT primers (Invitrogen). Quantitative PCR was performed using Taqman Gene Expression Assays (Applied Biosystems) with FAM-labeled probes for *ING4* (Hs00211773_m1), *IL8* (Hs00174103_m1), *IL6* (Hs00174131_m1), and *PGST2* (*COX2*) (Hs01573469_m1), and VIC-labeled probe for *GAPDH* (4310884E). Reactions were run using the Applied Biosystems 7900HT Fast Real-Time PCR system. Data were analyzed by the ΔΔC_t_-method normalized to *GAPDH*. For the qPCR array experiment, T47D-pMIG and T47D-ING4 cells were treated with 0.1% DMSO (vehicle control) or 50 ng/ml PMA for 4 hours in full growth media and RNA was isolated. One µg of total RNA was converted into cDNA using the RT2 First Strand Kit (SABiosciences-Qiagen). The qPCR reactions were performed using the 96-well Human NF-κB signaling targets RT^2^ Profiler PCR arrays (SABiosciences-Qiagen) and data were analyzed using the qPCR array analysis software from SABiosciences, according to the manufacturer’s directions.

### Gene Expression Profile Datasets

The GDS806 [44] and GSE3521/GPL887 [45] datasets containing gene expression profiles of 60 and 45 primary breast tumors samples, respectively, were retrieved from Gene Expression Omnibus (www.ncbi.nih.gov). The Netherlands Cancer Institute 295 (NKI295) dataset [46] containing gene expression profiles of 295 primary breast tumor samples was downloaded from the NKI website (http://bioinformatics.nki.nl/data.php).

### Statistical Analysis

Relationship between tumor pathologic features and molecular markers or between molecular markers was analyzed using Fisher’s Exact test. A dot plot was used to graph gene expression levels in each tumor. An unpaired 2-tailed student t-test was used to determine statistical significance. Kaplan-Meier survival plots were generated using GraphPad Prism (GraphPad Software, San Diego, CA). Receiver-operating characteristic (ROC) analysis was performed to determine the optimal cutoff point for categorization of ING4-low vs ING4-high expression. The log rank test was used to calculate statistical significance between the survival curves. *P*-values <0.05 were considered statistically significant.

## Results

### Monoclonal ING4 Antibody Generation

**Figure 1:**
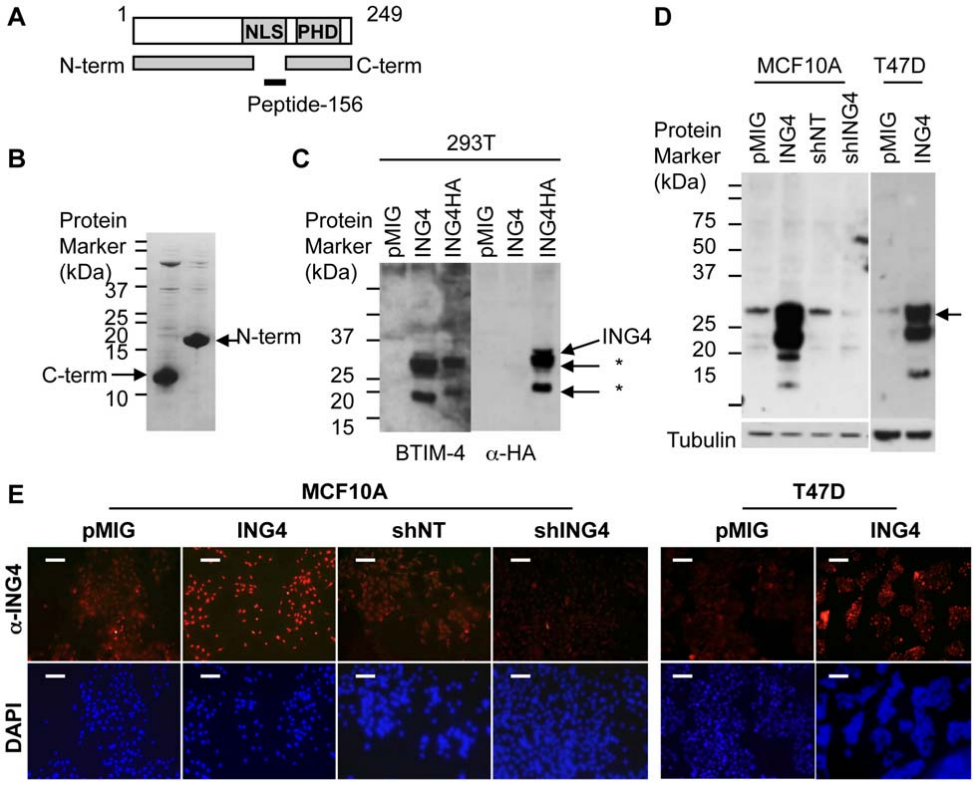
Generation and characterization of a monoclonal anti-ING4 antibody. (**A**) Schematic diagram of ING4 protein containing a nuclear localization signal (NLS) and a plant homeodomain (PHD). Stick figures represent the ING4 fragments that were used to immunize mice. (**B**) N-terminal (AA 5–147) and C-terminal (AA 173–249) recombinant fragments of ING4 purified from bacteria visualized by SDS-PAGE gel stained with Coomassie Blue. (**C**) Detection of ING4 and HA-epitope tagged ING4 overexpressed in 293T cells by Western blot using BTIM-4 and anti-HA monoclonal antibody. * denotes smaller ING4-derived protein species recognized by the antibody. (**D**) Western blot analysis of ING4 protein expression using BTIM-4 antibody in MCF10A breast epithelial cells and T47D breast cancer cells containing pMIG (the vector control), ING4 (ING4 overexpression), shNT (non-targeting shRNA control), or shING4 (shRNA targeting ING4). Tubulin antibody was used as a loading control. (**E**) MCF10A cells transduced with pMIG (the vector control), ING4 (ING4 overexpression), shNT (non-targeting shRNA control), or shING4 (shRNA targeting ING4), and T47D cells transduced with pMIG (the vector control) or ING4 (ING4 overexpression) were immunostained with BTIM-4 anti-ING4 antibody (red) and visualized using fluorescent microscopy. 4′,6-Diamidino-2-phenylindole (DAPI) was used to stain individual cell nuclei (blue). White scale bars represent 100 µm.

We have shown that the *ING4* gene is deleted in 16.5% of breast tumors, suggesting a tumor suppressive role in a subset of breast cancer [26]. In order to evaluate ING4 protein expression in breast tumors, we first generated a monoclonal antibody that recognized endogenous ING4 protein with specificity. We immunized mice with N-terminal (AA 5–147) or C-terminal (AA 173–249) recombinant ING4 protein fragments produced in bacteria and also with a synthetic peptide corresponding to amino acids 156–178 of ING4 (Figure 1A diagram and 1B). The N-terminal ING4 protein injection generated antibodies that recognized ING4 with specificity, one of which was designated BTIM-4. The BTIM-4 antibody detected full-length ING4 protein and ING4 epitope-tagged with hemagglutinin (HA) at the C-terminal end, overexpressed in 293T cells (Figure 1C). Both BTIM-4 and anti-HA antibodies detected additional ∼26 kDa and 17 kDa species which may represent degradation products of overexpressed ING4 (asterisks in Figure 1C). BTIM-4 antibody did not cross-react with the other ING family member proteins, ING1, ING2, or ING5, overexpressed in 293T cells, but did detect the mouse ING4 protein that shares 99% amino acid identity with the human ING4 protein (data not shown). The C-terminal ING4 protein injection failed to produce antibody specific to ING4 (data not shown), possibly due to the fact that the C-terminal fragment contains the PHD finger motif conserved among the ING family members and other transcription factors [35], [36]. The synthetic peptide injection generated antibodies that recognized overexpressed ING4 but with high background non-specific bands (data not shown). The BTIM-4 antibody was characterized further and referred to as anti-ING4 antibody herein.

### Detection of Endogenous ING4 Protein by anti-ING4 Antibody in MCF10A and T47D Cells

We tested whether anti-ING4 antibody detected endogenous ING4 protein in MCF10A and T47D cells by Western blot. MCF10A cells are a normal immortalized breast epithelial cell line with two copies of the *ING4* gene. T47D cells are a breast cancer cell line that contains only one copy of the *ING4* gene due to a defined deletion on chromosome 12 [23], [26], providing an example of “ING4 low” expressing cells. Consistent with this, T47D cells contained 10-fold less amount of *ING4* transcripts compared to MCF10A cells, normalized to the amount of *GAPDH* transcripts in each cell line (Figure S1A). The Western blot analysis of the cell lines showed that the anti-ING4 antibody detected a range of ING4 protein expression levels reflective of the relative mRNA expression in each cell line (Figure 1D).

We next performed immunofluorescent staining of ING4 in the MCF10A and T47D cell lines using the anti-ING4 antibody. The results showed nuclear and cytosolic staining of ING4 in both MCF10A and T47D vector control cells, indicating the antibody could detect endogenous levels of ING4 (Figure 1E and S1B). MCF10A and T47D cells overexpressing ING4 showed increased nuclear staining of ING4, compared to their respective vector control cells, whereas MCF10A cells with the ING4 knock-down construct showed diminished ING4 staining compared to the shNT control (Figure 1E and S1B). We increased the photographic exposure time for the T47D cell images in order to visualize the endogenous ING4 protein staining in T47D-pMIG cells, comparing it to the overexpressed ING4 protein staining in T47D-ING4 cells (Figure S1B). These results supported the sensitivity of the antibody in detecting different amounts of the ING4 protein.

### ING4 Immunohistochemical Staining of Breast Tumor Samples and Correlation with Tumor Features

**Figure 2:**
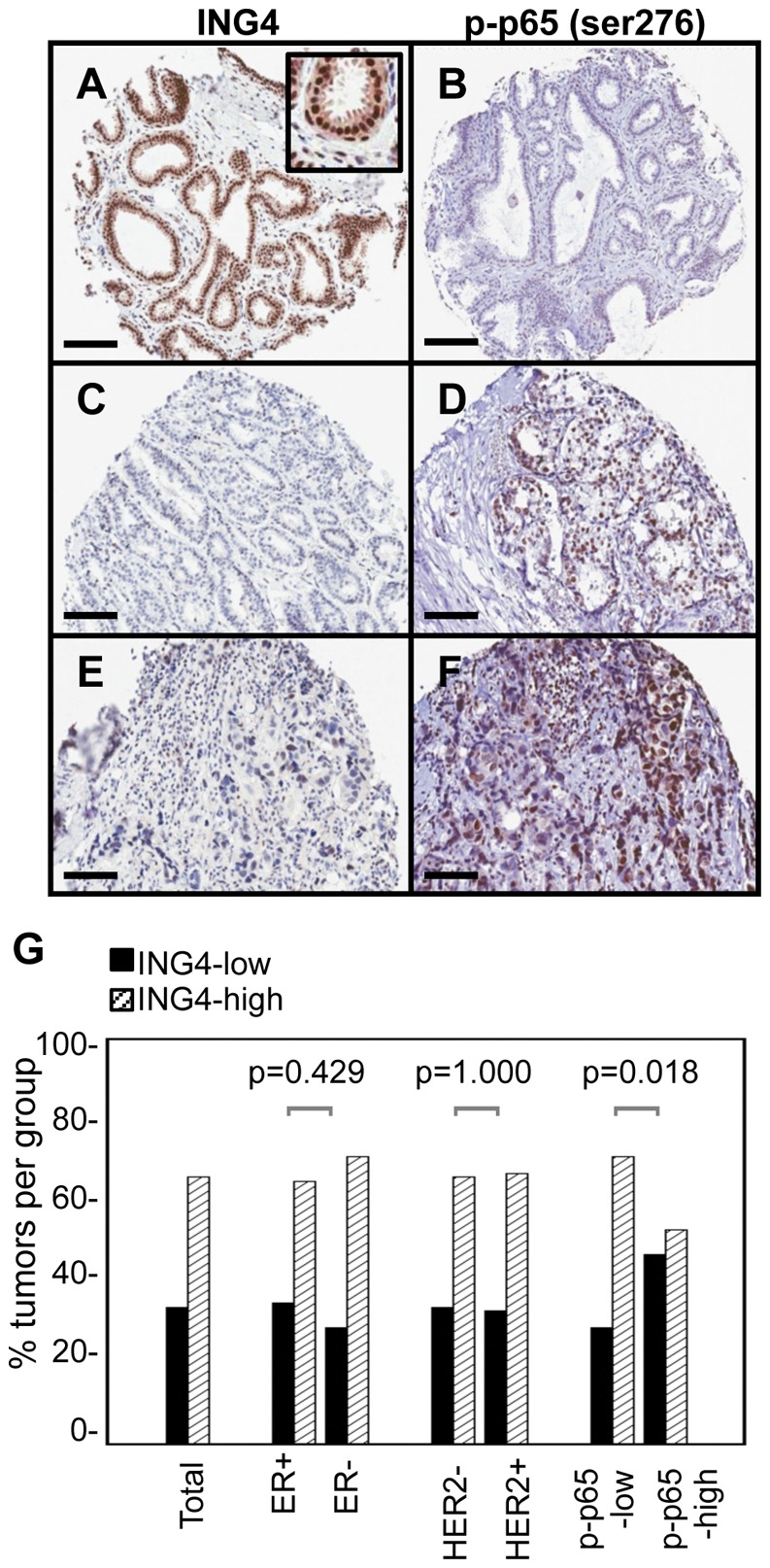
Inverse correlation between ING4 and p-p65/RelA (Ser276) antibody staining in breast tumor samples. (**A,B**) Hyperplastic breast tissue with moderate atypia from a ductal carcinoma in situ case, showing ING4 score +3 and p-p65 score 0. Inset is a higher magnification image of the region showing ING4 staining in luminal and basal epithelial cells and in stroma cells. (**C,D**) Invasive ductal carcinoma with ING4 score 0 and p-p65 score +2. (**E,F**) Grade 3 invasive ductal carcinoma with ING4 score +1 and p-p65 score +3. (bars, 100 µm). (**G**) Low ING4 expression is more prevalent in the tumors that express high levels of p-p65. Number of ING4-low (solid bars, IHC score <1.5) and ING4-high (slanted-line bars, IHC score ≥1.5) tumors, presented as a percentage of total number of tumors in each subgroup. Total (n = 227, 77 ING4-low, 150 ING4-high), ER+ (n = 156, 55 ING4-low, 101 ING4-high), ER− (n = 63, 18 ING4-low, 45 ING4-high), HER2- (n = 197, 67 ING4-low, 130 ING4-high), HER2+ (n = 27, 9 ING4-low, 18 ING4-high), p-p65-low (n = 166, 48 ING4-low, 118 ING4-high), and p-p65-high (n = 62, 29 ING4-low, 33 ING4-high). *P* values were determined by Fisher’s Exact test.

We proceeded to evaluate ING4 protein expression with the anti-ING4 antibody in breast tumor samples using immunohistochemistry on tumor tissue microarrays (TMAs). Nuclear staining of ING4 in tumor samples was scored on a scale of 0 to +3: intense and uniform nuclear staining was assigned +3 (Figure 2A) and no staining was assigned 0 (Figure 2C). A normal to slightly hyperplastic breast tissue section taken from a ductal carcinoma in situ (DCIS)-only case showed distinct nuclear ING4 staining in both luminal epithelial and myoepithelial cells within the ductal structure (Figure 2A inset). Stromal cells also stained for nuclear ING4 but with less intensity compared to epithelial cells. Normal breast tissue sections showed identical staining pattern and intensity for ING4 as the section sample shown in Figure 2A (data not shown).

We assigned an ING4 IHC score for each tumor sample by averaging the scores between “double” and “triple punches” on TMAs and defined less than 1.5 (<1.5) scores as “low ING4” and greater than or equal to 1.5 (≥1.5) scores as “high ING4.” We observed that 77 tumors among 227 tumors scored <1.5, constituting 34% of tumor samples that expressed low levels of ING4 (Table 1). We then correlated ING4 IHC scores with pathologic features including histologic subtype, tumor size, BRE grade, and lymph node status (see Table 1). All 6 DCIS cases showed +3 score for ING4 (Table 1), indicating that this non-invasive form of breast cancer expressed ING4 at a level well detectable by IHC. ING4-low tumors were comparably prevalent between invasive ductal carcinoma and lobular carcinoma (32% vs 30%; Table 1), suggesting a tumor suppressive role of ING4 in breast cancers rising from both ductal and lobular structures. Low ING4 expression was more frequently found in tumors that were large in size (≥2 cm in diameter, 41%), high grade (grade 2 and 3, 40–41%), and lymph node-positive (51%, Table 1; see bold font). Although we could not assign statistical significance likely due to the small cohort size, these data showed a consistent trend that more advanced breast tumors expressed low levels of ING4. These results suggested that down-regulation of ING4 may contribute to breast cancer progression.

**Table 1:** Tumors with low ING4 protein staining frequently have advanced tumor features.

Pathologicfeature	Subcategory	ING4<1.5	ING4≥1.5	*p*
		77 (**34%)**	150 (66%)	
Histologic subtype	DCIS*	0	6 (**100**%)	−
	IDC†	51 (32%)	108 (68%)	1
	ILC‡	6 (30%)	14 (70%)	
Size (diameter)	<2.0 cm	8 (28%)	21 (72%)	0.207
	≥2.0 cm	47 (**41%)**	68 (59%)	
BRE grade§	Grade 1	8 (27%)	20 (71%)	0.284
	Grade 2	29 (**40%**)	43 (60%)	
	Grade 3	17 (**41%**)	25 (59%)	
Lymph node status	N0	23 (35%)	43 (65%)	0.143
	> N0	19 (**51%)**	18 (49%)	

* DCIS, ductal cell carcinoma in situ;

† IDC, invasive ductal carcinoma;

‡ ILC, invasive lobular carcinoma;

§ BRE, Bloom, Richardson, Elston-Ellis grading.

### Low ING4 Expression Correlates with High Levels of Phosphorylated p65/RelA in Breast Tumors

We next correlated ING4 protein expression with the molecular subtype markers, ER and HER2. ING4-low tumors were equally prevalent between ER+ and ER-negative tumors (35% ER+ vs 29% ER-negative, Figure 2G), as was the case between HER2+ vs HER2-negative tumors (33% HER2+ vs 34% HER2-negative, Figure 2G). In addition, we did not observe an increased frequency of ING4-low tumors in any of the four molecular subtypes (luminal A, luminal B, basal-like, and HER2; data not shown). These results suggested that down-regulation of ING4 expression may be an independent event not related to the ER or HER2 status of the tumor.

We next tested whether low ING4 expression correlated with NF-κB activation in breast tumors by staining the TMAs with an antibody against the p65/RelA subunit phosphorylated at the amino acid residue serine 276 (p-p65/RelA), an activated form of NF-κB [47], [48]. Neither normal breast tissue nor the six DCIS samples showed nuclear p-p65/RelA IHC staining, suggesting that p-p65-high represents aberrant NF-κB activation in breast cancer (data not shown).

We scored nuclear p-p65/RelA staining on a scale of 0 to +3 (Figure 2B, D, F), averaged the scores between double and triple punches of each sample, and defined <1.5 scores as “p-p65-low” and ≥1.5 scores as “p-p65-high.” We then evaluated the relationship between p-p65/RelA and ING4 expression in breast tumor samples and found that 47% of p-p65-high tumors expressed low levels of ING4, compared to 29% of p-p65-low tumors (p = 0.018, Figure 2G). Two examples of the tumors that showed low ING4 and high p-p65 levels are shown in Figure 2C–F. These data indicated a statistically significant correlation between low ING4 and high p-p65 levels. Furthermore, we found that ING4-low/p-p65-high tumors were more frequently lymph node-positive (57%), compared to tumors with the other expression level makeups (27–39%, Table 2). These results suggested that phospho-activation of NF-κB may contribute to the high metastatic tendency of ING4-low tumors. Together, these results suggested that down-regulation of ING4 may foster phospho-activation of p65/RelA, resulting in aggressive breast cancer.

**Table 2:** Tumors with low ING4 and high p-p65 levels are frequently lymph node-positive.

Markers	Subcategories	LN−	LN+
(n = 103)		66 (64%)	37 (36%)
ING4<1.5	p-p65<1.5 (n = 28)	17 (61%)	11 (39%)
	**p-p65≥1.5 (n = 14)**	6 (43%)	8 **(57%)**
ING4≥1.5	p-p65<1.5 (n = 50)	35 (70%)	15 (30%)
	p-p65≥1.5 (n = 11)	8 (73%)	3 (27%)

### ING4 Inhibits p65/RelA Phosphorylation in T47D and MCF7 Cells

**Figure 3:**
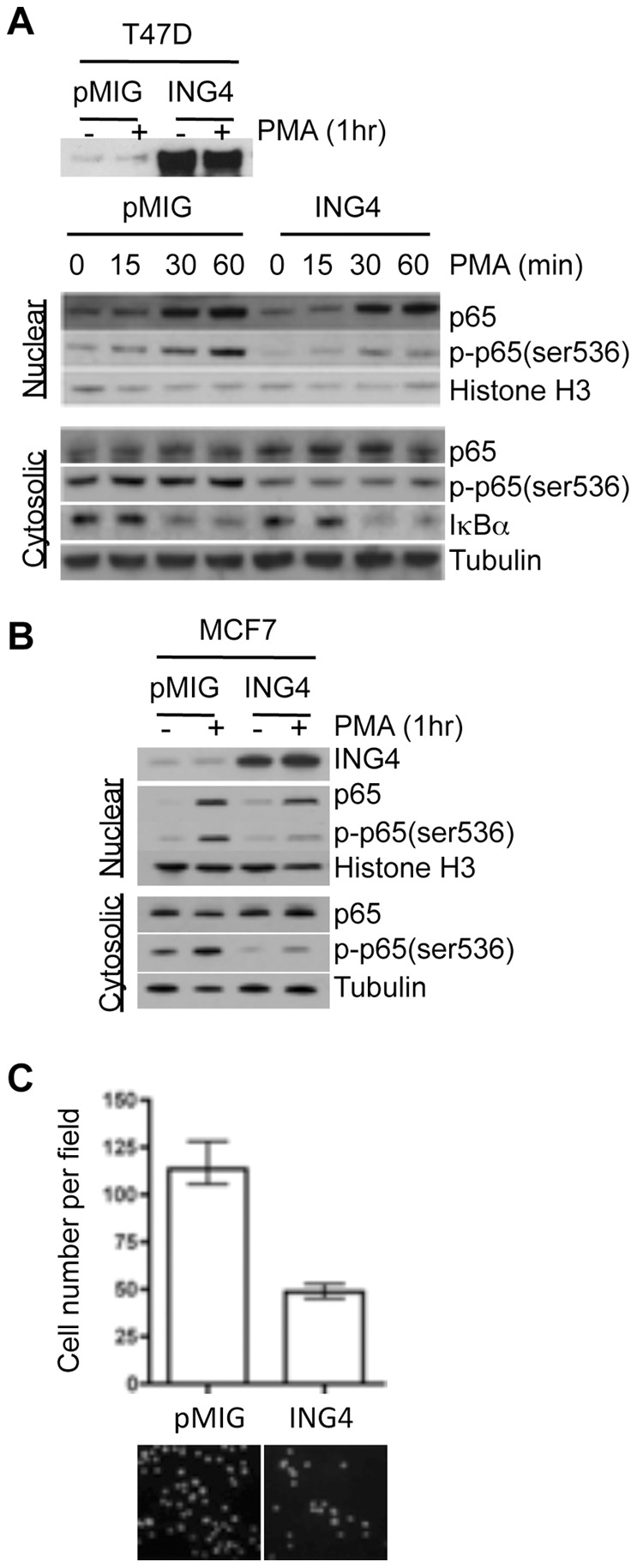
Reduced p65/RelA phosphorylation in breast cancer cells overexpressing ING4. (**A**) Western blot analysis of T47D cells expressing pMIG (the vector control) or ING4 (ING4 overexpression) for ING4, p65, p-p65 (Ser536), and IκBα. Cells were treated with PMA for 0, 15, 30 or 60 minutes prior to cell fractionation. (**B**) Western blot analysis of MCF7 cells expressing pMIG (the vector control) or ING4 (ING4 overexpression) for ING4, p65, and p-p65 (Ser536). Cells were treated with PMA for 60 minutes prior to cell fractionation. Histone H3 and Tubulin antibodies were used as loading controls for the nuclear and cytosolic fractions, respectively. (**C**) ING4 inhibits PMA-induced T47D cell invasion. Cells that invaded through a basement membrane matrix were stained with Hoechst dye and visualized under a fluorescent microscope (picture). Cell numbers were determined by averaging a minimum of 4 images per experiment from at least 6 independent experiments. *P* value was determined by *t*-test (p = 0.0011).

We tested whether ING4 protein expression levels affected phosphorylation of p65/RelA in breast cancer cell lines *in vitro*. We overexpressed ING4 in T47D and MCF7 breast cancer cells, treated cells with PMA to induce phosphorylation and nuclear localization of p65/RelA, and blotted for p-p65/RelA (Ser536), another activated form of p65/RelA [49], [50]. We could not assess the levels of p-p65/RelA (Ser276) by Western blot because the antibody was not suitable for Western blot analysis. On the other hand, the antibody against p-p65/RelA (Ser536) was not suitable for the IHC application. Thus, we used both forms of p-p65/RelA as the NF-κB activation markers, as was used in a previous study [48]. The Western blot results showed that the amounts of total p65/RelA protein in the cytosolic and nuclear fraction were comparable between the vector control and ING4 overexpressing cells (Figure 3A–B). These data indicated that ING4 overexpression did not affect the expression of p65/RelA or interfere with the nuclear localization of p65/RelA upon PMA activation. However, ING4 overexpressing cells contained measurably less p-p65/RelA (Ser536) in both cytosolic and nuclear fractions (2- to 5-fold), indicating that phosphorylation of p65/RelA at the amino acid residue serine 536 was inhibited by ING4 overexpression in T47D and MCF7 breast cancer cells (Figure 3A–B). These results were consistent with our observation in breast tumors by IHC that ING4 expression levels inversely correlated with phospho-activation of p65/RelA.

In mouse macrophages, ING4 was shown to positively regulate expression of the *NFΚBIA* gene that encodes Inhibitor of kappa B alpha (IκBα), thereby inhibiting NF-κB activation [33]. Therefore, we examined whether ING4 overexpression affected IκBα expression and found that the protein levels of IκBα were comparable between T47D-pMIG and T47D-ING4 cells (Figure 3A bottom panel), indicating that the mechanism of p65/RelA phosphorylation inhibition by ING4 may not involve the regulation of IκBα expression.

### ING4 Attenuates NF-κB-mediated Cell Invasion

We tested whether ING4 affected NF-κB-mediated cell invasion utilizing an invasion chamber assay. The results showed that ING4 overexpression attenuated PMA-induced cell invasion of T47D cells by more than 2-fold (Figure 3C) indicating that ING4 inhibited NF-κB-mediated cell invasion. These results suggested that breast tumors with low ING4 expression may be more invasive owing to NF-κB activation, consistent with the data that ING4-low/p-p65-high tumors were more frequently lymph node positive (Table 2).

### ING4 Attenuates NF-κB-mediated Gene Transcription

**Figure 4:**
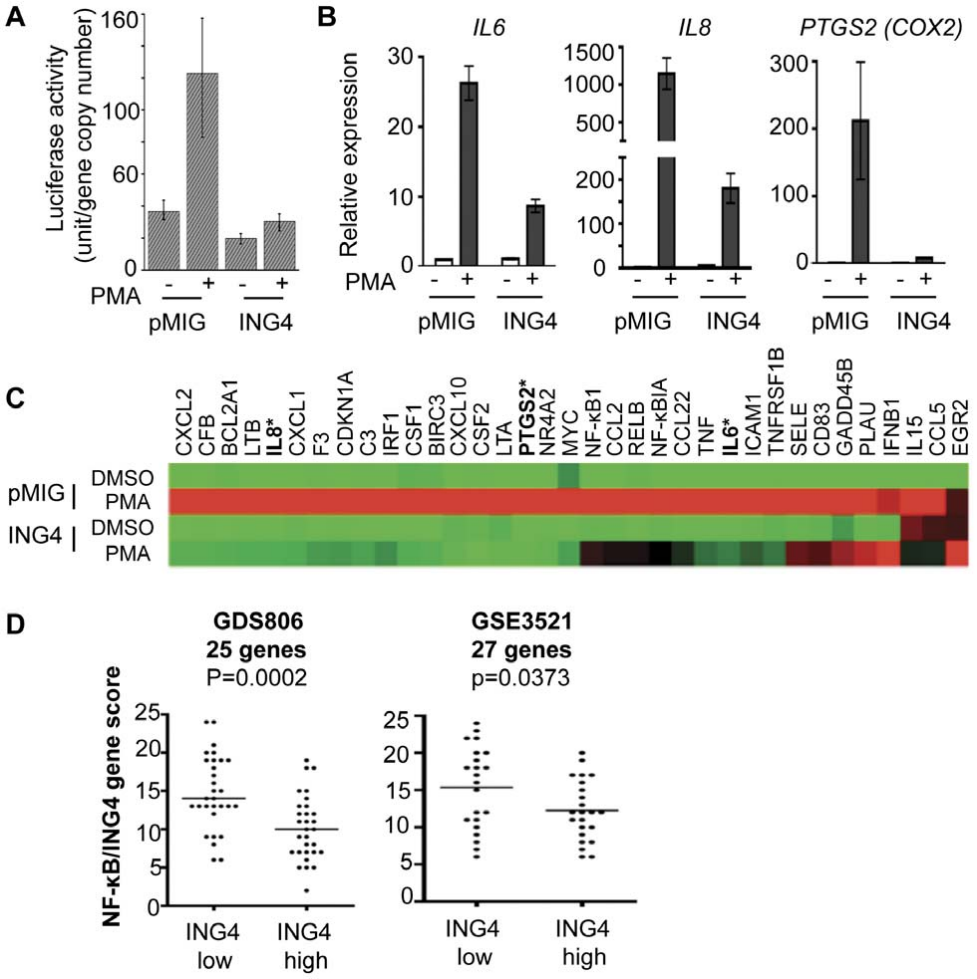
ING4 represses NF-κB-target gene expression. (**A**) ING4 represses expression of a luciferase reporter construct containing an NRE (NF-κB response element) promoter sequence in T47D cells. (**B**) ING4 represses expression of endogenous NF-κB-target genes, *IL6, IL8,* and *PTGS2*, evaluated by RT-qPCR. (**C**) Heat map of 35 NF-κB-target genes induced by PMA in T47D pMIG (vector) cells (red, 2^nd^ row). Twenty-seven of 35 genes were repressed by ING4 (green, 4^th^ row). Starred genes indicate those previously measured in single RT-qPCR assays. (**D**) ING4 transcript level inversely correlates with NF-κB-target gene expression in breast tumors in two independent gene expression profile data sets, GDS806 (n = 60) and GSE3521 (n = 45). GDS806 contained gene expression data for 25 of the 27 NF-κB-target genes and GSE3521 contained gene expression data for all 27 NF-κB-target genes.

In glioblastoma cells and melanoma-associated endothelial cells, ING4 was shown to repress expression of several NF-κB-target genes [20], [22], [25]. In order to determine whether ING4 regulated NF-κB target gene expression in breast cancer cells as well, we first used a luciferase reporter construct that contained the NF-κB response element (NRE) promoter in T47D cells. When the vector expressing cells, T47D-pMIG, were treated with PMA, the luciferase activity was induced by 3-fold (Figure 4A). ING4 overexpressing cells, T47D-ING4, however, failed to induce the luciferase reporter gene expression with PMA treatment, indicating that ING4 repressed NRE-dependent gene transcription (Figure 4A).

We next evaluated PMA-induced expression of endogenous NF-κB-target genes, *IL6, IL8,* and *PTGS2 (COX2)*, in T47D cells using RT-qPCR. The results showed that in PMA-treated T47D-pMIG cells, the expression of *IL6, IL8*, and *PTGS2* (*COX2)*, was induced by 26-, 1070-, and 212-fold, respectively (Figure 4B). In contrast, in PMA-treated T47D-ING4 cells, *IL6, IL8,* and *PTGS2 (COX2),* were induced by 8-, 35-, and 10-fold, respectively (Figure 4B). These results indicated that ING4 attenuated the expression of three endogenous NF-κB-target genes. Taken together with the luciferase reporter assay results, we concluded that ING4 attenuated NF-κB-mediated gene transcription in T47D breast cancer cells.

### ING4 Represses PMA-induced NF-κB-target Gene Expression

To evaluate the scope of NF-κB-target genes repressed by ING4, we compared the expression of 84 NF-κB-target genes in T47D-pMIG and T47D-ING4 cells with or without PMA treatment, using a qPCR array (for the complete gene list, see www.sabiosciences.com). In T47D-pMIG cells, PMA induced 35 NF-κB-target genes by more than 2-fold, ranging from 2- to 489-fold (Figure 4C and S2). These genes included CXC chemokines (*CXCL1, CXCL2, CXCL10*), CC chemokines (*CCL2, CCL5, CCL22*), interleukins (*IL6, IL8, IL15*), and NF-κB signaling molecules (*NFΚB1, NFΚBIA, RELB*). Among the 35 genes, 27 were repressed by ING4 by more than 2-fold in T47D-ING4 cells (Figure 4C and S2). These results indicated that more than 75% of PMA-induced genes were repressed by ING4 at least by 2-fold, suggesting a general repression mechanism of ING4. In addition, these data also suggested that breast tumors with low ING4 expression would express high levels of NF-κB-target genes.

### ING4 Expression Inversely Correlates with NF-κB-target Gene Expression in Primary Breast Tumors

To determine whether ING4-low breast tumors expressed high levels of the 27 NF-κB-target genes we identified above, we evaluated the GDS806 dataset [44]. The GDS806 dataset contained expression information for 25 out of the 27 NF-κB-target genes that we found repressed by ING4 in the qPCR array experiment, excluding the *C3* and *CSF1* genes. We compared the expression of 25 genes between ING4-low and ING4-high tumors by assigning an “ING4/NF-κB gene score” to each tumor. The “ING4/NF-κB gene score” of each tumor was calculated by first either assigning 0 for each NF-κB-target gene if the expression level was equal to or lower than the median expression of the gene within the dataset (no activation) or assigning +1 point for each gene if the expression level was greater than the median expression of the gene within the dataset (activation), followed by adding the points of a given set of genes, in this case, 25 genes. The results showed that ING4-low tumors had a mean ING4/NF-κB gene score of 14.8, whereas ING4-high tumors had a mean gene score of 10.2 (p = 0.0002, Figure 4D), indicating that ING4-low breast tumors expressed high levels of NF-κB-target genes compared to ING4-high tumors.

We examined an independent breast tumor gene expression dataset, GSE3521/GPL887, which contained the information on all 27 NF-κB-target genes repressed by ING4 [45]. We found the same correlation that ING4-low tumors had higher ING4/NF-κB gene scores in the GSE3521 data set, validating our observation with the GDS806 data set (p = 0.037, Figure 4D). These results demonstrated that ING4 expression levels inversely correlate with NF-κB-target gene expression in human primary breast tumor samples, supporting the function of ING4 in the repression of NF-κB-target genes.

### An ING4/NF-κB Gene Signature is Associated with Reduced Disease-free Survival in Breast Cancer

**Figure 5:**
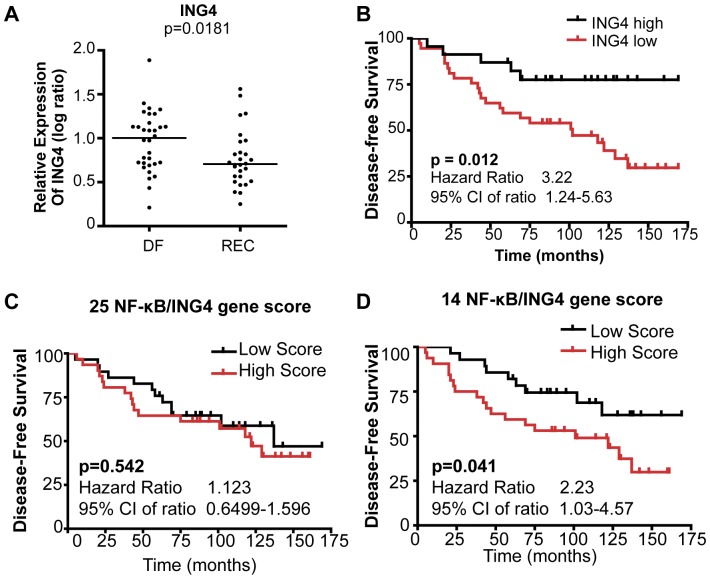
Low *ING4* mRNA expression and ING4-regulated NF-κB-target gene signature are associated with reduced disease-free survival in the GDS806 dataset. (**A**) Dot plot analysis of ING4 transcript levels in primary breast tumors from patients who remained disease-free (DF, n = 32) and from those who recurred (REC, n = 28) from the GDS806 dataset (n = 60). (**B**) Kaplan-Meier analysis of disease-free survival based on ING4 transcript level from the GDS806 dataset. CI, confidence interval. Kaplan-Meier analysis of disease-free survival based on NF-κB-target gene signatures (**C**) 25-gene or (**D**) 14-gene signature.

We next examined the relationship between low ING4 expression and breast tumor recurrence using the GDS806 dataset [44]. The dataset contained gene expression profiles of primary tumor samples from patients with ER-positive breast tumors, who remained disease-free (n = 32, median follow-up of 10 years) or who recurred (n = 28, median time to recurrence of 4 years) with adjuvant tamoxifen treatment. We compared the *ING4* transcript levels between the disease-free and recurrent tumor cohorts using a dot plot and found that primary tumors from patients who later recurred contained significantly reduced *ING4* transcript levels (p = 0.0181, Figure 5A). We did not find any statistical difference in the expression levels of the other four ING family members (ING1, ING2, ING3, and ING5) between disease-free and recurrent patient tumor samples, highlighting a unique relationship between ING4 expression and tumor recurrence (Figure S3).

We used Receiver-operating characteristic (ROC) analysis to determine the optimal cut point to group ING4-low vs ING4-high tumors for the GDS806 dataset and performed Kaplan-Meier analysis. The results showed that patients with ING4-low tumors had more than three times the rate of recurrence and significantly reduced disease-free survival (Figure 5B, HR = 3.22, 95% CI 1.24–5.63, log-rank *P* = 0.012), demonstrating that low ING4 expression was associated with reduced disease-free survival in ER+ breast cancer. We also analyzed an independent dataset, NKI295 [46], and found that low ING4 expression was associated with reduced disease-free survival (Figure S4, HR = 1.55, 95% CI 1.03–2.21, log-rank P = 0.036), validating that low ING4 expression is associated with reduced disease survival in breast cancer.

We next investigated whether high expression of ING4-regulated NF-κB-target genes was associated with reduced patient survival using the GDS806 dataset. We first analyzed the 25 genes as an ING4/NF-κB gene signature (Figure S2, 27 NF-κB-target genes repressed by ING4 at least by 2-fold, excluding *C3* and *CSF1* genes) and evaluated whether the ING4/NF-κB gene scores correlated with patient survival. The results showed no difference in disease-free survival between patients with high and low 25-ING4/NF-κB gene scores (Figure 5C). We next selected a 14 gene subset of the NF-κB-target genes based on the 4-fold or higher repression by ING4 (Figure S2) and examined whether the expression levels of these genes correlated with patient survival. The results showed that high 14-ING4/NF-κB gene scores were significantly associated with reduced disease-free survival (HR = 2.23, 95% CI 1.031–4.57, p = 0.0413, Figure 5D), indicating that the high expression of ING4/NF-κB-target genes recapitulated the association between low ING4 expression and reduced disease-free survival. Nine individual genes in the 14-gene subset showed a trend toward a correlation with reduced disease-free survival (data not shown), suggesting that each gene may contribute to the association of the collective 14-ING4/NF-κB gene signature with disease-free survival. These results support the idea that the tumor suppressor function of ING4 may involve the repression of a selective NF-κB-target gene set.

## Discussion

In a previous study, we reported that the *ING4* gene was deleted in 16.5% of breast tumors [26]. In this study, we showed that 34% of breast tumors expressed relatively low levels of the ING4 protein, implicating a tumor suppressive role for ING4 in a larger subset of breast cancer. In addition, breast tumors expressing low levels of the ING4 protein were frequently high grade and lymph node positive. Low ING4 expression has been associated with high-grade tumors and poor prognosis in other cancers including glioma, melanoma, and hepatocellular carcinoma [20], [28], [29]. Thus, it appears that down-regulation of ING4 may promote tumor progression in cancers originating from different tissue types.

Previously we showed that the *ING4* gene deletion was more prevalent in HER2+ tumors [26]. Considering that gene deletion may account for one of the mechanisms that result in low protein expression, we had expected that a higher percentage of HER2+ tumors would express low levels of the ING4 protein. On the contrary, we found that low ING4 expression was equally prevalent in HER2+ and HER2-negative tumors, suggesting no apparent link between the gene deletion rate and low protein expression. Neither the molecular basis for the elevated *ING4* gene deletion rate in HER2+ breast tumors nor the epigenetic mechanisms of ING4 down-regulation in breast tumors are known.

We found that low ING4 expression correlated with high levels of phosphorylated p65/RelA (p-p65/RelA), an activated form of NF-κB, in breast tumors, suggesting that down-regulation of ING4 may foster NF-κB activation in breast cancer. Elevated NF-κB activity has been well documented in HER2+ breast cancer [8]. However, we found the association between low ING4 expression and high levels of p-p65/RelA in all tumors regardless of the HER2 status, suggesting that ING4 down-regulation may represent a mechanism of NF-κB activation independent of HER2/neu receptor signaling in breast cancer.

Consistent with the inverse relationship between ING4 and p-p65/RelA levels in breast tumors, over-expression of ING4 resulted in the inhibition of p65/RelA phosphorylation in the T47D and MCF7 breast cancer cell lines. These data suggest that ING4 may negatively regulate p65/RelA phosphorylation but the molecular mechanism is presently unclear. Given the fact that ING4 is a transcriptional regulator, it is possible that ING4 regulates the expression of kinases or phosphatases involved in p65/RelA phosphorylation. To date, we found that ING4 did not alter the expression of PKA, MSK1, and Pim-1 kinases that were previously shown to phosphorylate p65/RelA at serine 276 [2], [47], [51], or IKKα and IKKβkinases, previously shown to phosphorylate p65/RelA at serine 536 [49], [50] (data not shown). Thus, while our data currently show an inverse relationship between ING4 expression levels and p65/RelA phosphorylation *in vivo* and *in vitro*, it is not clear whether ING4 is directly involved in the regulation of p65/RelA phosphorylation.

We showed that low ING4 mRNA expression was associated with reduced disease-free survival in breast cancer patients in two independent datasets. Our study constitutes the first report of the clinical consequence of low ING4 expression in breast cancer. It is of note that ING4-low tumors in the GDS806 dataset had more than three times the recurrence rate with a Hazard Ratio (HR) of 3.22, while the NKI295 dataset showed 1.55 HR. The precise reason for this difference is not clear. The GDS806 dataset consists of the tumor cohorts that were matched for patient age, tumor size, grade, and metastasis, between the disease-free and recurrent patient groups [44]. Thus, the efficient stratification of patients by ING4 expression levels in this dataset may suggest that ING4 expression levels may serve as an independent factor predictive of aggressive tumors that recur at a faster rate. Another possibility is that since the patient cohort in the GDS806 dataset was treated with adjuvant tamoxifen therapy [44], low ING4 expression may indicate tamoxifen resistance and/or recurrence. This would be consistent with other studies showing that elevated NF-κB activity is associated with endocrine therapy resistance [13], [14]. However, whether low ING4 expression is related to tamoxifen resistance requires further investigation and validation in a larger tamoxifen-treated cohort. The lower HR value of 1.55 in NKI295 compared to the one in GDS806 may reflect differences in the patient demographics between the two cohorts. The NKI295 patient cohort consists of premenopausal women with the median age of 44 whereas the GDS806 cohort has a median age of 68 [46], suggesting that low ING4 expression may not stratify younger patients as effectively. Further investigation is needed to determine the influence of patient age and menopausal status on the association between ING4 and disease-free survival.

Our study showed that ING4 repressed the majority of NF-κB-target genes (>75% of 35 genes). However, only a subset among the ING4-repressed NF-κB-target genes correlated with poor patient survival. These results suggested that the tumor suppressor function of ING4 may be through the repression of a subset of NF-κB-target genes responsible for aggressive tumor behaviors. Consistent with this idea, many genes in the ING4/NF-κB gene signature have been implicated to have a role in aggressive breast cancer. For example, elevated expression of CXCL10 and its receptor CXCR3 has been associated with breast cancer metastasis [52]. CCL2 and CCL5 have been shown to promote tumor cell migration and invasion [53]. Up-regulation of PTGS2 (COX2) has long been documented in various cancers and explored as a therapeutic target [54]. In addition, pro-tumorigenic roles for TNF and IL-6 have also been well established [55]. In our gene expression dataset analysis, elevated expression of most single genes in the ING4/NF-κB gene signature did not significantly correlate with poor patient survival. However, collective high expression of the gene set correlated with reduced disease-free survival. Whether this points to a functional cooperation between the ING4/NF-κB genes resulting in aggressive disease is not clear. In this study, we surveyed a limited set of 84 NF-κB target genes using PMA as an agent to active NF-κB. Using other NF-κB activating agents such as TNF and surveying more extensive NF-κB-target gene sets may help to refine the ING4/NF-κB gene signature associated with aggressive breast cancer. In addition, as inflammation has emerged as a hallmark of cancer in general [4], [56], the ING4/NF-κB gene signature may represent one of the inflammatory gene signatures associated with tumor progression and aggressive disease in other cancer types.

We conclude that ING4 negatively regulates NF-κB in breast cancer. Consequently, down-regulation of ING4 results in NF-κB activation, leading to disease progression and poor patient outcome in breast cancer. Our study provides insight into the tumor suppressor function of ING4 in breast cancer and offers a molecular basis to explore the ING4/NF-κB pathway as a potential therapeutic target in order to block disease progression and improve patient survival.

## Supporting Information

Figure S1 ING4 mRNA expression and immunofluorescent staining of ING4 in the MCF10A and T47D cells expressing the ING4 overexpression construct or knock-down construct.
(TIFF)

Figure S2 Fold repression of 35 NF-κB-target genes by ING4.
(TIFF)

Figure S3 No correlation between transcript levels of the other ING family members, ING1, ING2, ING3, and ING5, with tumor recurrence.
(TIFF)

Figure S4 Low ING4 expression correlates with reduced disease-free survival in breast cancer patients in the NKI295 dataset.
(TIFF)
