# Supplementary figures and images for: Negative Regulation of NF-κB by the ING4 Tumor Suppressor in Breast Cancer
Source: PLoS One. 2012 Oct 4;7(10):e46823. doi: 10.1371/journal.pone.0046823 (PMC3464231; doi:10.1371/journal.pone.0046823)

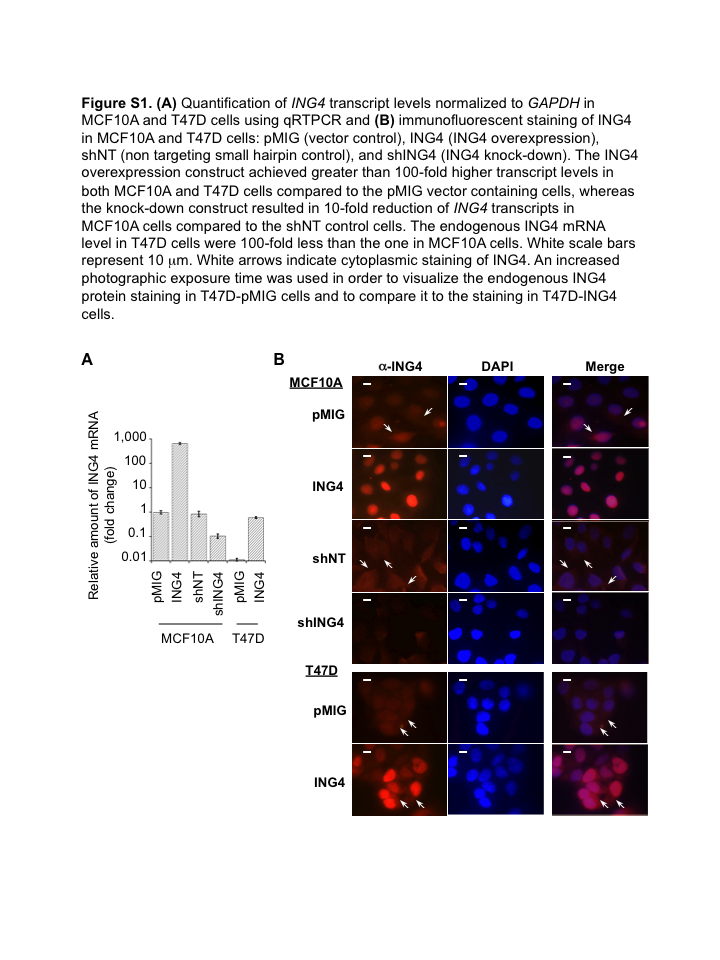

Supplement: Figure S1 — ING4 mRNA expression and immunofluorescent staining of ING4 in the MCF10A and T47D cells expressing the ING4 overexpression construct or knock-down construct. (TIFF) [file pone.0046823.s001.tiff]

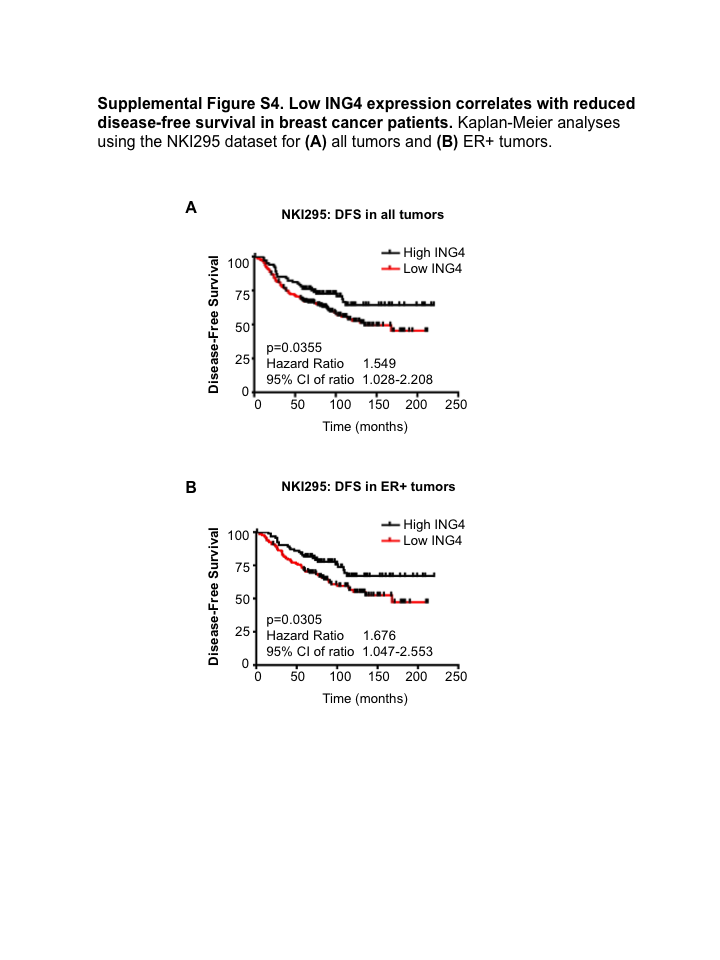

Supplement: Figure S4 — Low ING4 expression correlates with reduced disease-free survival in breast cancer patients in the NKI295 dataset. (TIFF) [file pone.0046823.s004.tiff]
